# Influenza and SARS-CoV-2 Co-infections in California, USA, September 2020–April 2021

**DOI:** 10.3201/eid2711.211129

**Published:** 2021-11

**Authors:** Kyle R. Rizzo, Cora Hoover, Seema Jain, Monica Sun, Jennifer F. Myers, Brooke Bregman, Deniz M. Dominguez, Allison Jacobsen, Garrett J. Jenkins, Tamara Hennessy-Burt, Erin L. Murray

**Affiliations:** California Department of Public Health, Richmond, California, USA

**Keywords:** California, co-infections, coronavirus disease, COVID-19, influenza, respiratory infections, SARS-CoV-2, severe acute respiratory syndrome coronavirus 2, United States, viruses, zoonoses

## Abstract

During September 1, 2020–April 30, 2021, the California Department of Public Health, Richmond, California, USA, received 255 positive influenza molecular test results that matched with severe acute respiratory syndrome coronavirus 2 molecular test results; 58 (23%) persons were co-infected. Influenza activity was minimal in California, and co-infections were sporadic.

The public health community anticipated widespread co-circulation of influenza and severe acute respiratory syndrome coronavirus 2 (SARS-CoV-2), the virus that causes coronavirus disease (COVID-19), during the 2020–21 influenza season. However, influenza activity in California was unusually low (*1*). The California Department of Public Health (CDPH; Richmond, California, USA) matched positive influenza test results with SARS-CoV-2 test results to assess the occurrence of influenza and SARS-CoV-2 co-infections in California.

## The Study

California laboratories and medical providers must report all positive and nonpositive (i.e., negative, inconclusive, or invalid) SARS-CoV-2 laboratory results to their local health jurisdictions (LHJs) (*2*). For influenza, only positive results that can be submitted electronically by laboratories are reportable. Most data are reported directly to CDPH’s web-based platform, California Reportable Diseases Information Exchange (CalREDIE). CalREDIE assigns electronic laboratory reports a unique identifier, personID, that can be used to link the same person across different disease reports. CalREDIE is used by 59 of California’s 61 LHJs for disease tracking and reporting. Two LHJs, Los Angeles and San Diego, which represent one third of California’s population, do not use CalREDIE directly; we excluded data from those LHJs.

We matched positive molecular influenza test results reported during September 1, 2020–April 30, 2021, with positive and nonpositive molecular SARS-CoV-2 test results to identify co-infections. We matched positive influenza results with nonpositive SARS-CoV-2 results to determine whether persons infected with influenza were negative for SARS-CoV-2 or were potentially not tested for SARS-CoV-2. We deduplicated all positive influenza tests results and excluded antigen test results.

**Figure 1:**
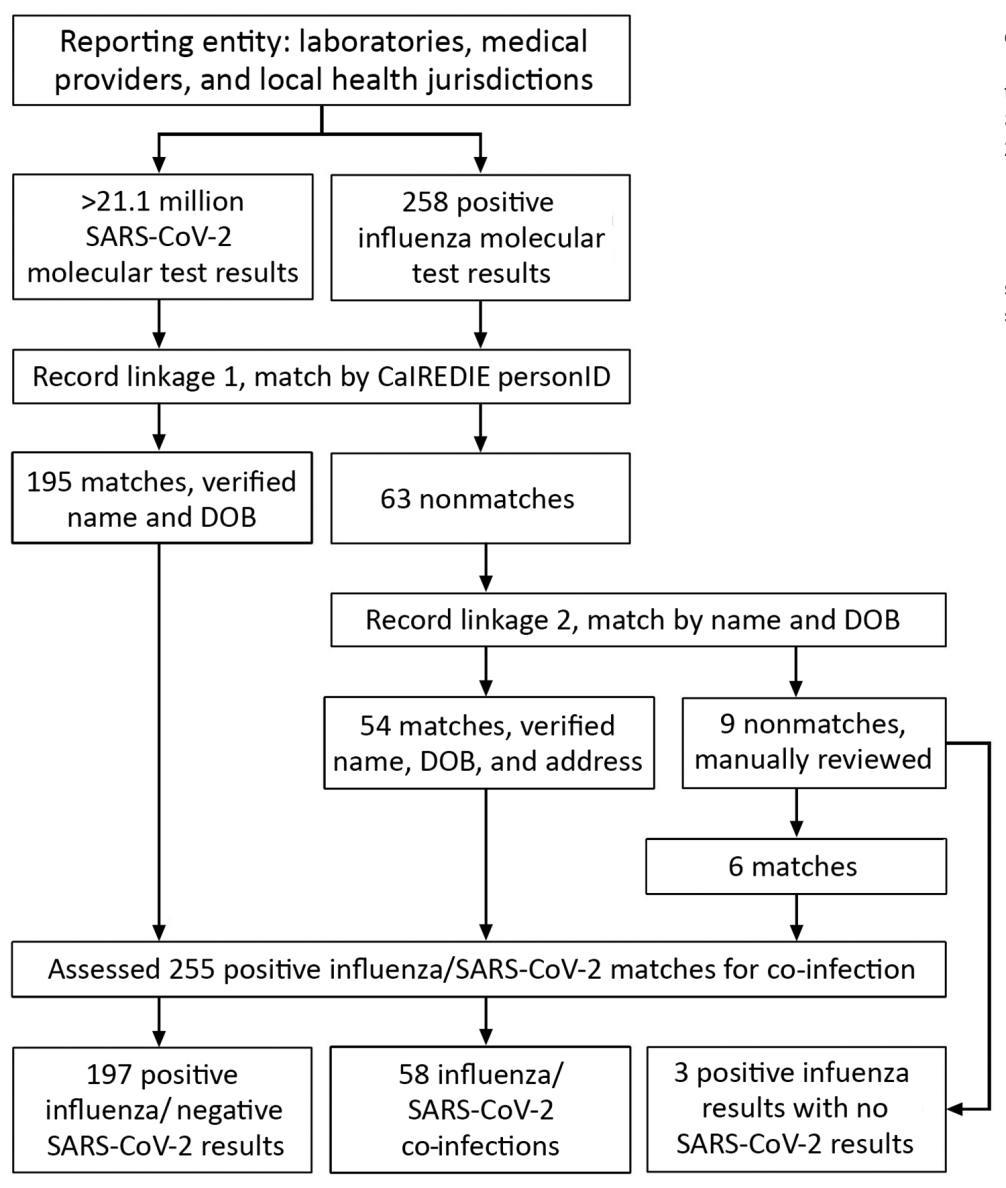
Matching process of influenza and SARS-CoV-2 molecular test results submitted to CalREDIE, California, USA, September 1, 2020–April 30, 2021. CalREDIE, California Reportable Diseases Information Exchange; DOB, date of birth; personID, CalREDIE individual identification cod SARS-CoV-2, severe acute respiratory syndrome coronavirus 2

We matched laboratory results first using CalREDIE personID, then by name and date of birth, and finally by manual record review if positive influenza results did not match to SARS-CoV-2 results by personID or name and date of birth (Figure 1). If a person had both positive and nonpositive SARS-CoV-2 results within 7 days of a positive influenza result, we used the positive SARS-CoV-2 result in the analysis. Persons with both positive influenza and SARS-CoV-2 test results with ≤7 days between specimen collection dates met criteria for influenza and SARS-CoV-2 co-infection. We analyzed co-infection data by week of illness onset and geographic distribution. We summarized co-infected persons by age, race and ethnicity, sex, hospitalization, and survival status. We completed all analyses using SAS version 9.4 (SAS Institute, http://www.sas.com). This study received a nonresearch determination from the California Committee for the Protection of Human Subjects.

**Figure 2:**
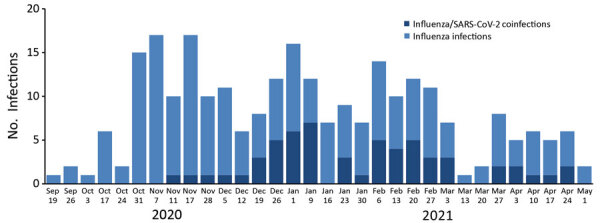
Number of influenza infections (n = 258) and co-infections with SARS-CoV-2 (n = 58) by week of onset, California, USA, September 1, 2020–April 30, 2021. SARS-CoV-2, severe acute respiratory syndrome coronavirus 2.

CDPH received 258 positive influenza test results during September 1, 2020–April 30, 2021, and >21.1 million SARS-CoV-2 total test results. Among positive influenza results, 255 (99%) matched with a SARS-CoV-2 test result (positive or nonpositive). From these matches, 58 (23%) persons were co-infected with influenza and SARS-CoV-2 and 197 (77%) were positive for influenza and negative for SARS-CoV-2 (Figure 1). Co-infections occurred sporadically in California beginning in mid-November 2020 (Figure 2). At least 1 positive influenza result was received from 35 (59%) of 59 reporting LHJs, and ≥1 co-infections were identified in 21 (36%) LHJs throughout all regions in California. Among the 258 persons with positive influenza tests, 170 (66%) had influenza B and 88 (34%) influenza A. Influenza B was predominant (n = 39; 67%) among co-infected persons. Fifty-two (90%) co-infected persons had influenza and SARS-CoV-2 test specimens collected on the same date. 

Age distribution among co-infected persons was 5 (9%) who were 0–17 years, 23 (40%) 18–49 years, 12 (21%) 50–64 years, and 18 (31%) ≥65 years of age. Twenty-two (38%) persons were female and 35 (60%) were male; sex was unknown for 1 (2%) co-infected person. The racial/ethnic distribution of co-infected persons was 20 (34%) Latino, 20 (34%) White, 5 (9%) Asian, 3 (5%) African American, 1 (2%) American Indian or Alaskan Native, 1 (2%) Native Hawaiian or Pacific Islander, 2 (3%) other, 2 (3%) multirace, and 4 (7%) unknown. Among 28 (48%) co-infected persons with available hospitalization status data, 11 (39%) were hospitalized. Five (9%) co-infected persons died, including 2 who were hospitalized; all who died were >50 years of age.

## Conclusions

Influenza activity was minimal during the 2020–21 influenza season in the United States and Northern Hemisphere, after low levels were reported in the Southern Hemisphere during the 2020 season there (*3*,*4*). Only 258 positive influenza test results were reported to CDPH during September 1, 2020–April 30, 2021, in contrast to the >1.77 million COVID-19 cases reported by the 59 LHJs included in this analysis. The low numbers of influenza infections in this report are consistent with California sentinel laboratory data and national trends (*1*,*3*). In addition, influenza activity in California during 2020–21 was at a historic low based on clinical sentinel laboratory data collected from 2009–2020 (*5*). Less than 1% of specimens tested were positive for influenza at California clinical sentinel laboratories throughout the 2020–21 influenza season, compared with peaks in influenza specimen positivity of 24%–41% in prior seasons. Overall, 58 (23%) of 255 persons with a positive influenza test result and a matching SARS-CoV-2 test result met our case definition for a co-infection.

Multiple factors likely account for the 2020–21 influenza season trends we observed in California. Travel was substantially affected by shelter-in-place policies, and reduced travel might have interrupted traditional influenza transmission patterns in which travelers carry influenza viruses between regions. Adopting COVID-19 mitigation practices, including social distancing, wearing face coverings, and closing schools and businesses, might also have helped prevent the transmission of influenza in communities. Factors such as viral interference might have contributed to the uncharacteristically low influenza activity reported (*6*). It is not yet clear how much influenza vaccination contributed to the minimal levels of influenza activity reported. Preliminary data from the Centers for Disease Control and Prevention indicate that overall, ≈53% of US adults had received the 2020–21 seasonal influenza vaccine by January 2021, compared with 45% of adults who had received the 2019–20 vaccine by January 2020. However, vaccination percentages were lower among children, especially Black and Hispanic children, and pregnant women in 2021 (*7*).

The first limitation of our analysis is that the lack of influenza laboratory data from Los Angeles and San Diego LHJs likely underestimates the number of influenza infections and co-infections with SARS-CoV-2 in California. However, Los Angeles and San Diego LHJs reported similarly low levels of influenza activity, and thus, including data from those LHJs is unlikely to have changed the main findings of this analysis (*8*,*9*). We could not assess whether medical providers and patients sought influenza testing during this surveillance period as routinely as they did in years past because nonpositive influenza test results are not reportable to CDPH. It is possible that SARS-CoV-2 testing was prioritized over influenza virus testing, and infrequent or inaccessible influenza testing might have contributed to underestimates of influenza transmission in California. Finally, we did not require that test results reported by laboratories and medical providers undergo confirmatory testing by public health laboratories for inclusion in this analysis.

Ongoing public health surveillance is needed to assess the burden of SARS-CoV-2 infection and interactions with other respiratory viruses, including influenza. Healthcare providers should consider testing patients for influenza and SARS-CoV-2 on the basis of the local epidemiology of these infections and public health guidance. Safe and effective vaccines are available throughout the United States to prevent against both influenza and COVID-19 (*10*,*11*). Healthcare providers should encourage influenza and COVID-19 vaccination as a primary prevention strategy for all community members, especially among persons of color and low-income residents, who are disproportionately affected by both diseases.
